# Application of convolutional neural networks for automated segmentation and classification in esophageal diseases

**DOI:** 10.3389/fmed.2026.1742019

**Published:** 2026-03-06

**Authors:** Liangpeng Pu, Xiao Wang, Shanshan Yan, Shuaishuai Zhuang, Xiaopu He

**Affiliations:** 1 The First School of Clinical Medicine of Nanjing Medical University, Nanjing, China; 2 The First Affiliated Hospital with Nanjing Medical University, Nanjing, China

**Keywords:** artificial intelligence, convolutional neural network, deep learning, endoscopic images, esophageal lesions

## Abstract

**Objective:**

To develop a convolutional neural network (CNN) framework for the automated segmentation and classification of esophageal lesions in endoscopic images.

**Methods:**

(1) Lesion localization was performed using a Region-based Convolutional Neural Network (R-CNN). (2) A dual-stream Esophageal Lesion Network (ELNet) was developed to classify images into four diagnostic categories. (3) Lesion segmentation was carried out using an ensemble of three U-Net architectures.

**Results:**

The dual-stream ELNet achieved a classification accuracy of 92.14%, with 97.1% specificity and 88.74% sensitivity. The segmentation module based on U-Net attained an overall accuracy of 95.54% and a lesion segmentation sensitivity of 82.89%. The dual-stream ELNet consistently outperformed single-stream baseline networks, and the integrated segmentation-with-classification architecture demonstrated enhanced adaptability across diverse lesion types.

**Conclusion:**

The proposed CNN framework enables accurate, robust, and simultaneous classification and segmentation of esophageal endoscopic lesions, exhibiting high performance and clinical potential.

## 1 Introduction

Esophageal mucosal lesions—ranging from benign conditions such as esophagitis and gastric heterotopia to precancerous dysplasia and invasive carcinoma—are commonly encountered in clinical practice. Among these, early esophageal cancer arises through progressive mucosal alterations, often without overt symptoms until advanced stages. Consequently, timely and accurate detection of mucosal abnormalities is critical for early intervention, improved prognosis, and reduced mortality (1).

Current diagnostic modalities for esophageal lesions include computed tomography (CT), barium swallow radiography, and endoscopy. Of these, white-light endoscopy remains the gold standard due to its ability to directly visualize lesion morphology, color, surface pattern, and spatial extent in real time (2, 3). Accurate identification of lesion boundaries and characteristics in endoscopic images enables clinicians to formulate precise treatment strategies, including targeted biopsies or endoscopic resection (4). However, visual interpretation of endoscopic findings is inherently subjective and highly dependent on the endoscopist’s experience, leading to substantial inter-observer variability—particularly in distinguishing subtle mucosal changes associated with early neoplasia (5).

**FIGURE 1:**
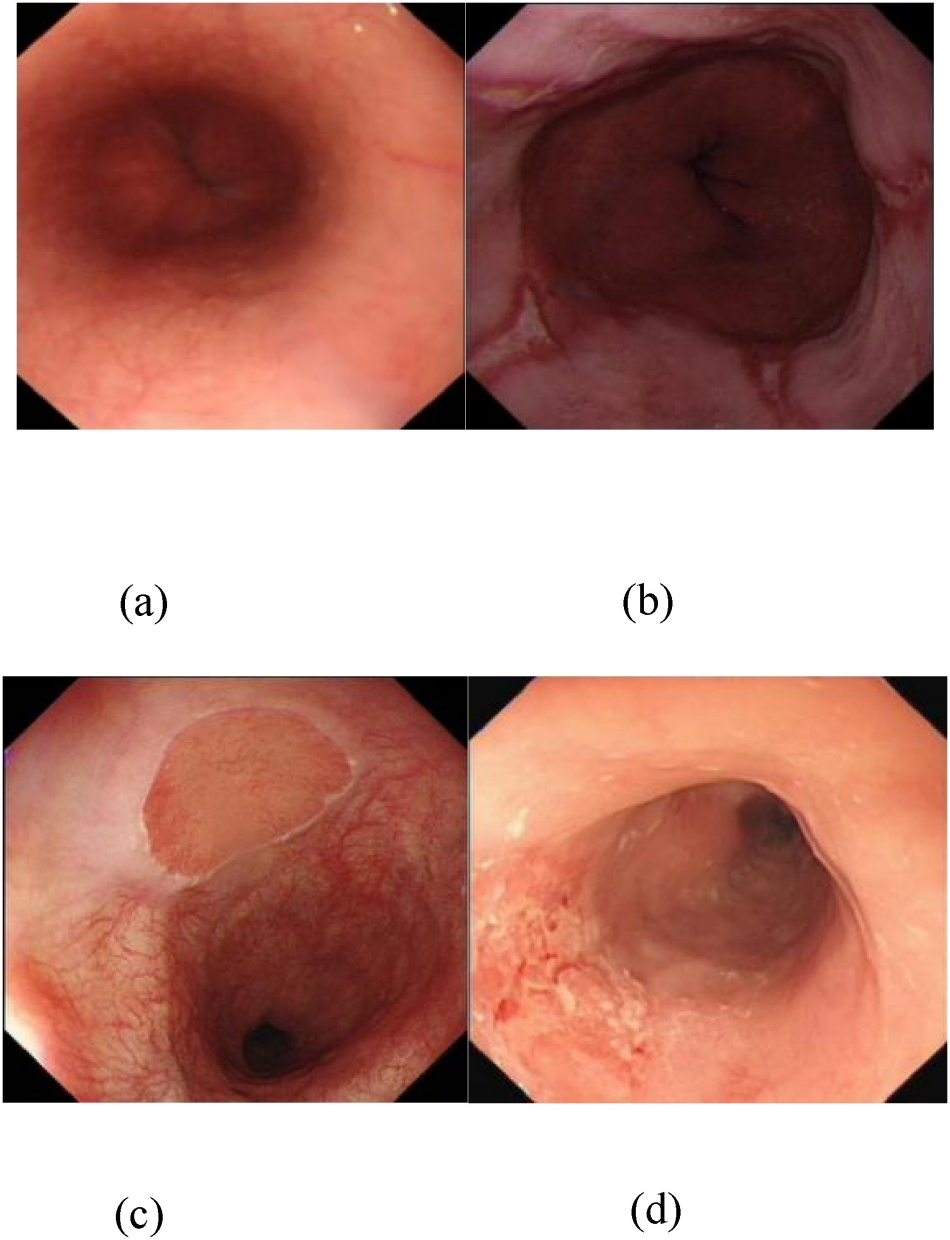
Four types of esophageal images. **(a)** Normal type indicates no lesions in the esophageal image. **(b)** Inflammatory lesion type features red-white banded mucosa. **(c)** Heterotopic mucosa lesion type is characterized by well-defined boundaries between normal areas and intraepithelial lesion regions. **(d)** Neoplastic lesion type exhibits mucosal irregularity, vascular irregularity, and absence of vascular patterns.

In recent years, computer vision and artificial intelligence (AI) have emerged as transformative tools in medical image analysis, offering objective, reproducible, and scalable solutions for disease detection and characterization (6, 7). By integrating multimodal neuroimaging data with cerebrospinal fluid biomarkers, artificial intelligence has significantly improved diagnostic accuracy for Alzheimer’s disease, demonstrating considerable potential in this field (8). In the context of esophageal endoscopy, AI-assisted systems hold significant promise not only for improving diagnostic accuracy but also for enabling population-level cancer screening, supporting less-experienced endoscopists, and standardizing clinical decision-making? (9). The differentiation among common esophageal conditions—including esophageal cancer, esophagitis, gastric heterotopia, and normal mucosa—relies heavily on visual cues such as texture, color heterogeneity, vascular patterns, and architectural distortion? (10). As illustrated in Figure 1, these features vary distinctly across pathologies:

- Figure 1a shows a normal esophageal mucosa with regular vascular patterns;
- Figure 1b depicts esophagitis, characterized by erythematous, friable mucosa with alternating red–white striations;
- Figure 1c demonstrates gastric heterotopia, featuring well-demarcated patches of ectopic gastric-type epithelium;
- Figure 1d reveals esophageal cancer, marked by irregular surface topography, loss of normal architecture, and abnormal microvascular networks—hallmarks of dysplastic transformation? (10).

Accurate classification of these four categories aids in risk stratification and therapeutic planning, while precise lesion segmentation—based on color, shape, and spatial extent—provides critical guidance for defining resection margins during endoscopic therapy.

Despite these opportunities, automated analysis of esophageal endoscopic images remains challenging due to factors such as non-uniform illumination, specular reflections, mucus artifacts, and intra-class variability in lesion appearance. Traditional computer-aided diagnosis (CAD) systems rely on handcrafted features—such as Gabor-filtered textures or color histograms—combined with shallow classifiers like support vector machines (SVMs)? (11). While promising in controlled settings, these approaches are limited by their dependence on manual feature engineering and poor generalizability across diverse imaging conditions.

Deep learning, particularly convolutional neural networks (CNNs), has revolutionized medical image analysis by automatically learning hierarchical representations directly from raw data. A notable advance was achieved in AI-based glaucoma screening with the development of an efficient hybrid framework (VGG16 combined with ML classifiers), which attained 97.7% accuracy using 40% fewer parameters? (12). Recent studies have demonstrated the efficacy of CNNs in esophageal lesion detection: Li et al. reported a deep CNN system achieving 89.7% sensitivity, 98.5% specificity, and 98.2% accuracy in identifying high-risk lesions across a cohort of over 3,000 patients? (13). Similarly, Hussein et al. developed a CNN framework to assist clinicians in lesion localization and biopsy decision-making? (14). Nevertheless, most existing models focus either on classification or segmentation, often neglecting the synergistic potential of joint analysis. Moreover, direct full-image segmentation is prone to false positives due to the abundance of non-lesion structures (e.g., normal mucosa, bubbles, and reflections) in esophageal scenes, while classification without spatial localization lacks the necessary context for pixel-level delineation.

To address these limitations, we propose a clinically inspired, cascaded framework that mirrors the diagnostic workflow of expert endoscopists: localization → classification → segmentation. First, a Faster R-CNN module identifies suspicious regions of interest, effectively filtering out irrelevant background and reducing interference. Subsequently, a dual-stream network (ELNet) performs fine-grained classification of the localized region into one of four diagnostic categories. Finally, the classification result dynamically routes the input to a corresponding category-specific U-Net for precise lesion segmentation. This design not only aligns with real-world clinical reasoning but also ensures computational efficiency—only one segmentation branch is activated during inference. By integrating task-specific modules in a structured pipeline, our approach achieves robust, end-to-end assistance for early esophageal lesion diagnosis under real-world white-light endoscopy conditions.

## 2 Materials and methods

**FIGURE 2:**
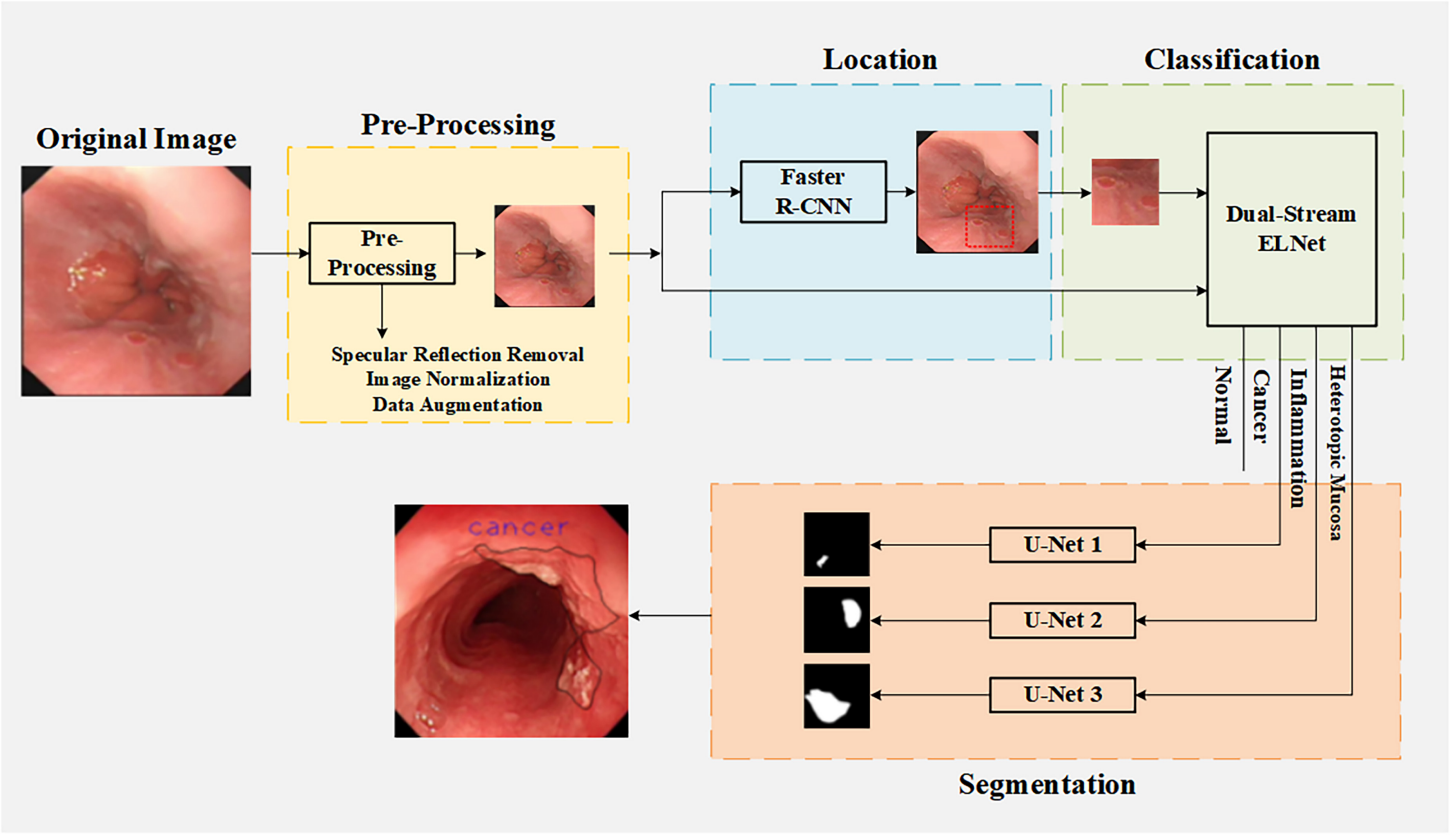
Main framework of the proposed method for esophageal lesion classification and segmentation.

The overall workflow of our framework is illustrated in Figure 2, which depicts a clinically inspired, cascaded pipeline comprising four sequential stages: (1) image preprocessing, (2) lesion localization via Faster R-CNN, (3) four-category classification using dual-stream ELNet, and (4) lesion-type-specific segmentation with dedicated U-Nets. This structured design mirrors the diagnostic reasoning of expert endoscopists and ensures computational efficiency by activating only one segmentation branch per inference.

The dataset comprised 789 white-light endoscopic esophageal images from 832 patients at The First Affiliated Hospital of Nanjing Medical University (May 2018–May 2019). Selection criteria included routine case analysis and standard endoscopic examination, excluding post-resection and poor-quality images. All images featured specialist-annotated pixel-level labels verified by pathological results, with ethical approval obtained.

This study used 10-fold cross-validation, with 80 % of images for training, 10 % for testing, and 10 % for validation to improve generalization and optimize parameters. All datasets were non-overlapping. Image statistics are shown in Table 1.

**TABLE 1:** Statistical distribution from the esophageal image database.

Dataset	Normal	Inflammation	Esophageal cancer	Heterotopic mucosa
Training	186	172	85	188
Validation	23	22	10	24
Testing	23	22	10	24
Total	232	216	105	236

### 2.1 Preprocessing of esophageal white-light endoscopic images

Preprocessing involves three steps: removing specular reflections from images, normalizing to reduce computational complexity, and applying data augmentation to prevent overfitting.

#### 2.1.1 Specular reflection removal

Endoscopic images often show specular reflections from the moist, smooth esophageal lining, appearing as white spots that may be mistaken for lesions. To address this issue, we implemented the algorithm proposed by Tchoulack S, which operates in streaming mode through dual-task processing (15).

First, two-dimensional histogram decomposition is employed to detect specular reflection, as shown in Equations 1, 2:


$$\text{m}=\frac{1}{3}\,\left(r+g+b\right)$$ (1)

$$\text{S}=\left\{ \begin{array}{c} \frac{1}{2}\,\left(2\,r-g-b\right)=\frac{3}{2}\,\left(r-m\right)\,i\,f\,\left(b+r\right)>2\,g\\ \frac{1}{2}\left(r+g+-2b\right)=\frac{3}{2}\left(m-b\right)if\left(b+r\right)\geq 2g \end{array}\right.$$ (2)

Here, r, g, b are the red, green, blue channels; s is saturation; m is pixel intensity. Thresholds m_*max*_ and s_*max*_ enable specular reflection detection using a 2D histogram-based method. A pixel p is classified as belonging to a specular highlight when the following criterion in Equation 3 is met:


$$\left\{ \begin{array}{c} \text{m p}\geq \frac{1}{2}\,{\text{m}}_{\max}\\ \text{s p}\leq \frac{1}{3}\,{\text{s}}_{\max} \end{array}\right.$$ (3)

Parameters m_*max*_ and S_*max*_ represent the maximum values of m_*p*_ and s_*p*_ in the entire image. These thresholds were determined through empirical analysis of extensive esophageal endoscopic images.

**FIGURE 3:**
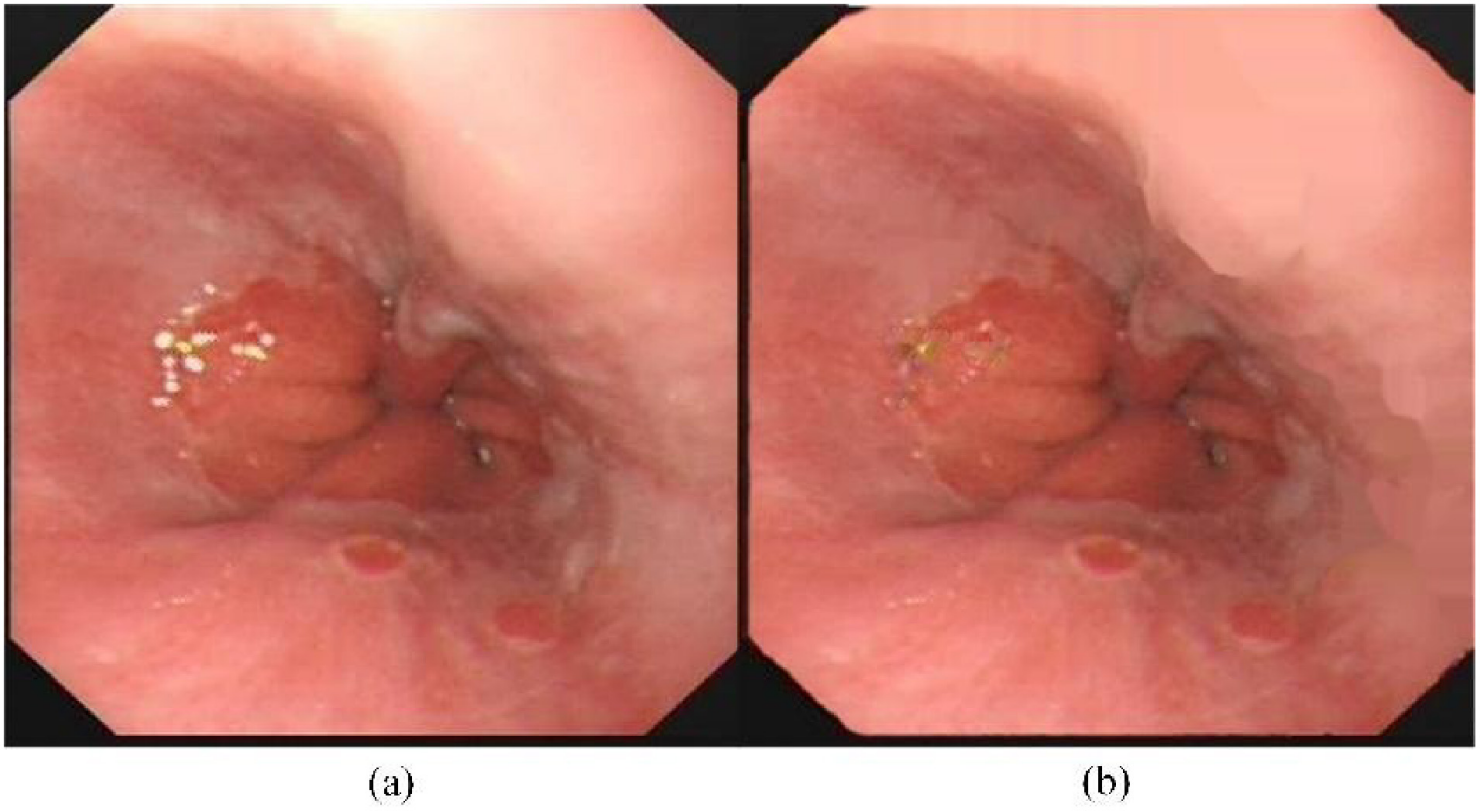
Typical images before and after specular reflection removal. **(a)** Original image **(b)** Image after specular reflection removal.

Subsequently, the Navier-Stokes algorithm is utilized to perform inpainting correction (16). Pixels detected as specular reflection are replaced with the average value of adjacent pixels to smooth the corrected images. Figure 3 presents representative endoscopic images before and after the elimination of specular reflections.

#### 2.1.2 Image normalization

To reduce computation, images are downsampled to 512 × 512 via bilinear interpolation. For classification, lesion regions in the local stream are resized to 64 × 64.

For tri-channel esophageal endoscopic images, pixel values were normalized to the range (0, 1). The normalization formula is defined in Equation 4:


$$\text{y}=\frac{\text{x}-M\,i\,n\,V\,a\,l\,u\,e}{M\,a\,x\,V\,a\,l\,u\,e-M\,i\,n\,V\,a\,l\,u\,e}$$ (4)

Here, y is the normalized pixel value, with MaxValue and MinValue being the image’s maximum and minimum pixel values, and x the original pixel value.

#### 2.1.3 Data augmentation

Due to limited training samples, data augmentation was applied to reduce overfitting. The augmented dataset includes geometric transformations such as flipping, rotation, scaling, and translation, with parameters specified in Table 2.

**TABLE 2:** Data augmentation parameters.

Transformation type	Description
Rotation	Random rotation 0−360rotation angle
Flip	0 (No Flip) or 1 (Flip)
Stretch	Random stretching with scale factor between 1/1.6 and 1.6
Translation	Random shift between -10 and 10 pixels

### 2.2 Localization of esophageal lesions

The localization framework was architected upon the Faster R-CNN model, which integrates a Fast R-CNN, a Region Proposal Network (RPN), and a feature extraction network. For the purpose of spatial feature extraction, the feature extraction network leveraged the VGG16 architecture (17). The RPN generates lesion proposals using VGG16’s final convolutional feature map with sliding windows. Each window matches three multi-scale anchors at a 1:1 aspect ratio to prevent shape distortion. The regression layer refines bounding box coordinates, while the classification layer with Softmax evaluates anchor likelihoods. Fast R-CNN then verifies lesion presence in these regions.

Subsequently, the lesion bounding box’s center coordinates and dimensions are obtained. The localized region is then resized and input to Dual-stream ELNet’s local stream for classification.

### 2.3 Classification of esophageal lesions

**FIGURE 4:**
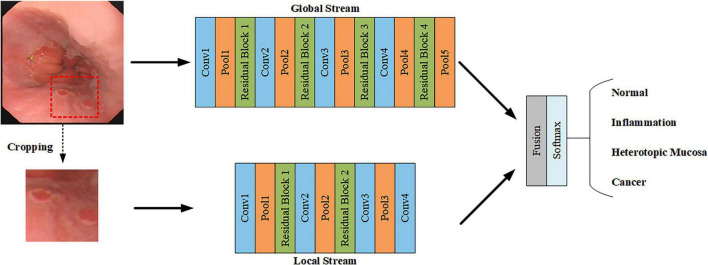
Structure of the dual-stream ELNet.

We developed dual-stream ELNet with local and global streams for esophageal lesion classification. The local stream extracts lesion color, texture, and shape features from four-type image patches (Normal, Inflammation, Ectopia, Cancer), while the global stream analyzes color contrast and size from entire images. Three lesion-type patches are obtained via Faster R-CNN, with normal patches from random cropping. The architecture is shown in Figure 4.

Table 3 details the Dual-stream ELNet architecture. The local stream contains 13 layers (10 convolutional, 3 pooling), with Conv 4 aligning output dimensions to the global stream. Each convolutional layer is followed by batch normalization and ReLU. The global stream has 21 layers (16 convolutional, 5 pooling), where all convolutional layers use stride 1 except Conv 1 (stride 2).

**TABLE 3:** Detailed design of the dual-stream ELNet for esophageal lesion classification.

Dual-stream ELNet							
Global stream				Local stream			
Layer		Kernel size, channel number	Output size	Layer		Kernel size, channel number	Output size
Data		-	512 × 512	Data			64 × 64
Conv 1		3 × 3, 64	256 × 256	Conv1		3 × 3,64	64 × 64
Pool 1		2 × 2, 64	128 × 128	Pool 1		2 × 2, 64	32 × 32
Residual block-1	Conv 1	1 × 1, 32	128 × 128	Residual block-1	Conv1	1 × 1,32	32 × 32
	Conv 2	3 × 3, 32	128 × 128		Conv2	3 × 3,32	32 × 32
	Conv 3	1 × 1, 64	128 × 128		Conv3	1 × 1,64	32 × 32
Conv2		3 × 3, 128	128 × 128	Conv2		3 × 3,128	32 × 32
Pool 2		2 × 2, 128	64 × 64	Pool 2		2 × 2,128	16 × 16
Residual block-2	Conv 1	1 × 1, 64	64 × 64	Residual block-2	Conv 1	1 × 1,64	16 × 16
	Conv 2	3 × 3, 64	64 × 64		Conv 2	3 × 3,64	16 × 16
	Conv 3	1 × 1, 128	64 × 64		Conv 3	1 × 1,128	16 × 16
Conv3		3 × 3, 256	64 × 64	Conv3		3 × 3,256	16 × 16
Pool 3		2 × 2, 256	32 × 32	Pool 3		2 × 2,256	8 × 8
Residual block-3	Conv 1	1 × 1, 128	32 × 32	Conv 4		1 × 1,512	8 × 8
	Conv 2	3 × 3, 128	32 × 32	-		-	-
	Conv 3	1 × 1, 256	32 × 32	-		-	-
Conv 4		3 × 3, 512	32 × 32	-			
Pool 4		2 × 2, 512	16 × 16	-		-	-
Residual block-4	Conv 1	1 × 1, 256	16 × 16	-		-	-
	Conv 2	3 × 3, 256	16 × 16	-		-	-
	Conv 3	1 × 1, 512	16 × 16	-		-	-
Pool 5		2 × 2, 512	8 × 8	-		-	-
Fusion		8 × 8, 1,024					
Softmax		4 Neurons					

**FIGURE 5:**
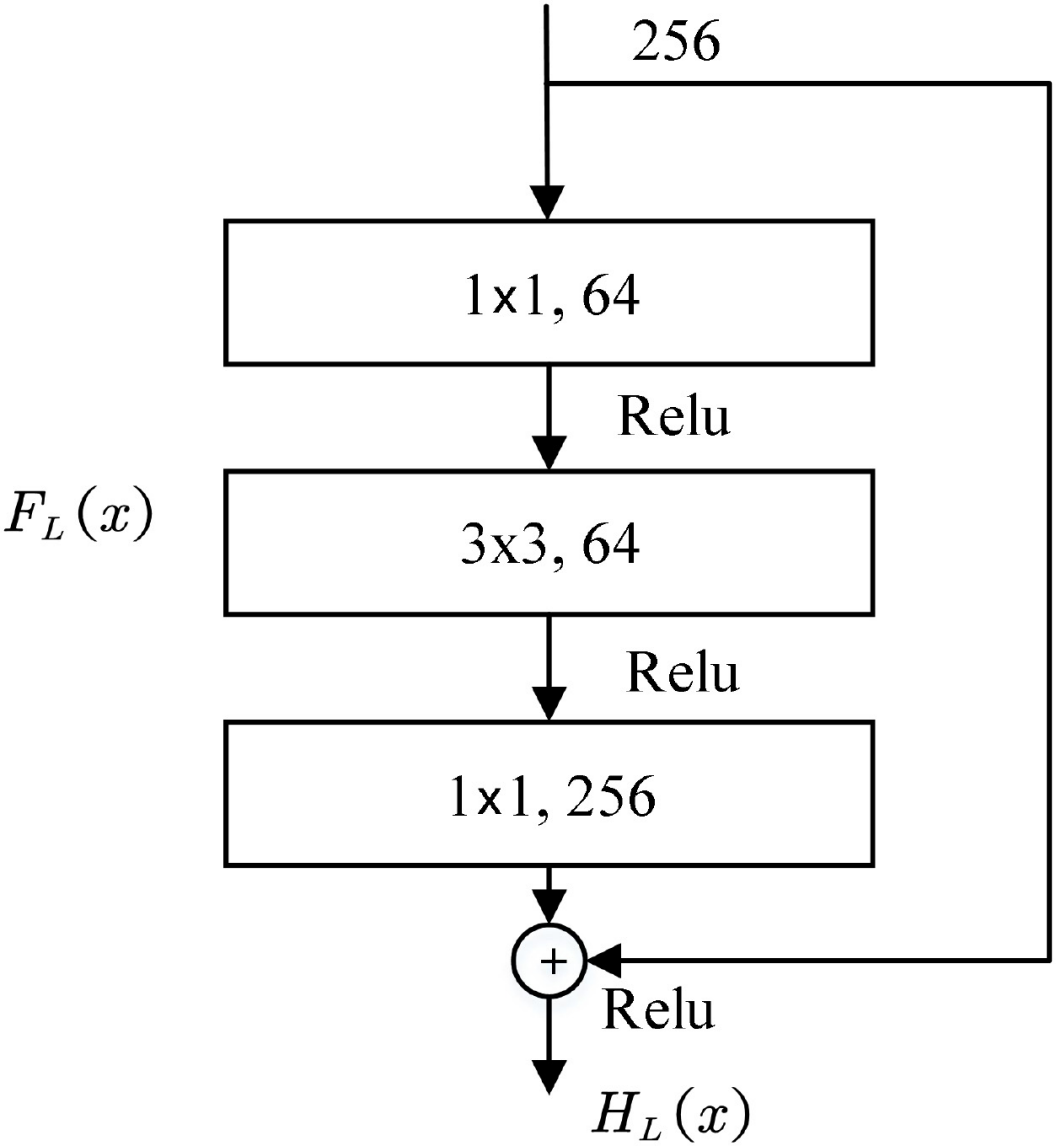
Construction block of the residual network.

The foundation of both local and global streams is built upon the ResNet-50 architecture established by He et al. (18). The introduction of skip connections in ResNet serves to combat the performance degradation problem in deep networks. The design of the residual block is presented schematically in Figure 5. This process is slightly cumbersome; therefore, for convenience, *x*serves as the building block *L^th^* input, *H*_*L*_ (*x*) denotes the building block *L^th^* transformation function, the expected output of the residual block is defined as *F*_*L*_ (*x*). The residual block is explicitly designed to compel the output to fit a residual mapping. In other words, this mapping is achieved through stacked nonlinear layers, which is mathematically represented in Equation 5:


$$F_{L}\,\left(x\right)=H_{L}\,\left(x\right)-x$$ (5)

Thus, the transformation of the building block is given by Equation 6:


$$H_{L}\,\left(x\right)=F_{L}\,\left(x\right)+x$$ (6)

The residual block uses 1 × 1 and 3 × 3 convolutional kernels, where the 3 × 3 layer extracts spatial features while the 1 × 1 layer reduces channels.

The pooling layer reduces computational load via down-sampling. As detailed in Table 2, data dimensions are down-sampled through pooling layers from 512 × 512 (global) and 64 × 64 (local) to 8 × 8 pixels.

A cascaded fusion method is used to integrate the data outputs from the global flow and local stream vectors (19). We define a fused feature map y, two feature maps x*^a^* and x*^b^*, and a fusion function y = f_*cat*_ (x*^a^*, x*^b^*), where y ∈ R^H′×W′×D′^, x^b^∈*R^H×W×D^*, x^*a*^∈*R^H×W×D^*, where W, H and D correspond to the width, height, and depth of the feature maps, respectively. y = f_*cat*_ (x*^a^*, x*^b^*) concatenates two features at identical spatial positions (i, j) along the channel dimension d as shown in Equation 7:


$$y_{i,j,d}=x_{i,j,d}^{a},y_{i,j,D+d}=x_{i,j,d}^{b}$$ (7)

where y∈*R*^H×W×2D^. The fused feature map measures 8 × 8 pixels, yielding 1,024 features. These features are flattened before input to the Softmax layer.

The Softmax layer normalizes the feature maps to values between 0 and 1, enabling the output vector y_*m*_ to signify the m-th class probability. The Softmax operation is expressed in Equation 8:


$$y_{m}=\frac{e^{\theta_{m}\,x}}{\sum_{m=1}^{4}e^{\theta_{m}\,x}}$$ (8)

where *x* denotes input neurons from the preceding layer, θ_*m*_ represents weight parameters for the m-th class, and y_*m*_ denotes the output probability for the m-th class.

The dual-stream ELNet employs cross-entropy loss as its objective function for accelerated training, with the loss function defined in Equation 9 (based on cross-entropy):


$$\mathrm{loss}=-\mathrm{mean}\,\left(y\cdot \log\left(\hat{y}\right)+\left(1-y\right)\cdot \log\left(1-\hat{y}\right)\right)$$ (9)

where y denotes the predicted output vector from the classification ELNet, and y represents the label vector.

### 2.4 Segmentation of esophageal lesions

Given the distinct texture, color, size, and shape characteristics of esophageal cancer, esophagitis, and esophageal gastric heterotopia lesions, we first developed the Segmentation Network with No Classification (SNNC) for unclassified lesion segmentation. However, intra-class variations impeded discriminative feature learning. To address this limitation, we propose the Segmentation Network with Classification (SNC) method, which employs Dual-stream ELNet for initial classification followed by type-specific segmentation, thereby improving lesion-type specificity. Our study implements SNC through three architecturally identical segmentation networks and provides comparative evaluation with SNNC.

Building upon the U-Net architecture, we established a segmentation network for esophageal lesions, which has shown promising performance in medical image segmentation (20). The typical U-Net architecture comprises a symmetric expanding path, a contracting path, and a bottleneck layer that intermediates between these two paths.

This study employs a U-Net with four contraction blocks for feature extraction from esophageal images. Each block contains a 2 × 2 max-pooling layer and two 3 × 3 convolutional layers. As shown in Table 4, the contracting path reduces input dimensions from 512 × 512 to 32 × 32 pixels. The expanding path performs pixel-wise binary classification through four expansion blocks, each comprising two 3 × 3 convolutional layers, a concatenation layer, and a deconvolution layer with stride 2. The contracting path’s input size matches the expanding path’s output size, with each output vector providing background/foreground probabilities.

**TABLE 4:** Architecture of U-Net for esophageal lesion segmentation.

Contracting path			Expanding path		
Layer	Kernel size, channel number	Output size	Layer	Kernel size, channel number	Output size
Data	-	512 × 512	Deconv 1	2 × 2, 512	64 × 64
Conv 1	3 × 3, 64	512 × 512	Concat 1	–, 1024	64 × 64
Conv 2	3 × 3, 64	512 × 512	Conv 1d	3 × 3, 512	64 × 64
Pool 1	2 × 2, 64	256 × 256	Conv 2d	3 × 3, 512	64 × 64
Conv3	3 × 3, 128	256 × 256	Deconv 2	2 × 2, 256	128 × 128
Conv4	3 × 3, 128	256 × 256	Concat 2	–, 512	128 × 128
Pool 2	2 × 2, 128	128 × 128	Conv 3d	3 × 3, 256	128 × 128
Conv 5	3 × 3, 256	128 × 128	Conv 4d	3 × 3, 256	128 × 128
Conv 6	3 × 3, 256	128 × 128	Deconv 3	2 × 2, 128	256 × 256
Pool 3	2 × 2, 256	64 × 64	Concat 3	–, 256	256 × 256
Conv 7	3 × 3, 512	64 × 64	Conv 5d	3 × 3, 128	256 × 256
Conv 8	3 × 3, 512	64 × 64	Conv 6d	3 × 3, 128	256 × 256
Pool 4	2 × 2, 512	32 × 32	Deconv 4	2 × 2, 64	512 × 512
Bottleneck			Concat 4	–, 128	512 × 512
Conv 9	3 × 3, 1,024	32 × 32	Conv 7d	3 × 3, 64	512 × 512
Conv 10	3 × 3, 1,024	32 × 32	Conv 8d	3 × 3, 64	512 × 512
–	–	–	Conv 9d	1 × 1, 2	512 × 512

The bottleneck layer contains two 3 × 3 convolutional layers. The U-Net follows each convolutional layer with a ReLU layer and a batch normalization layer. Loss is calculated using the binary cross-entropy function.

### 2.5 Implementation plan

This study details the implementation pipeline. The code used Python 3.6, TensorFlow r1.4, and CUDA 10.0 on an NVIDIA 2080TI GPU and i7-5930K CPU.

Models were trained for 10,000 iterations using mini-batch stochastic gradient descent with 0.9 momentum. The learning rate was initialized at 1 and progressively reduced until the loss plateaued.

The classification network used a batch size of 8 during training, while the three segmentation U-Nets used a batch size of 4. During testing, Dual-stream ELNet processed both 64 × 64 and 512 × 512 test images across four subgroups (normal, cancerous, heterotopic mucosa, inflammatory), with classified samples then routed to corresponding U-Nets: cancer to U-Net 1, inflammation to U-Net 2, and heterotopic mucosa to U-Net 3.

## 3 Results and evaluation methods

This study presents quantitative and qualitative results, evaluating: (1) agreement between algorithm and expert annotations (segmentation performance), and (2) classification accuracy of esophageal images. Experiments included: (1) assessing classification performance of local, global, and dual-stream ELNet; (2) implementing SNC to segment three lesion types, with quantitative and qualitative comparisons between SNNC and SNC segmentation results.

The classification performance was assessed using Receiver Operating Characteristic (ROC) curves, along with the metrics of accuracy, specificity, and sensitivity. Accuracy, specificity, and sensitivity were defined using Equations 10–12:


$$S\,E\,N\,S=\frac{T\,P}{T\,P+F\,N}$$ (10)

$$S\,P\,E\,C=\frac{T\,N}{F\,P+T\,N}$$ (11)

$$A\,C\,C=\frac{\left(T\,P+T\,N\right)}{\left(T\,P+T\,N+F\,P+F\,N\right)}$$ (12)

True positive (TP) corresponds to the count of positive images that are accurately identified.

True negative (TN) corresponds to the number of negative images correctly classified.

False positive (FP) corresponds to the number of instances where negative cases are incorrectly predicted as positive.

False negative (FN) corresponds to the number of instances where positive cases are erroneously classified as negative.

### 3.1 Classification of esophageal lesions

Table 5 compares the Local stream, Global stream, and Dual-stream ELNet, evaluating Accuracy, Specificity, and Sensitivity for lesion classification. The Dual-stream ELNet outperformed both subnetworks across all metrics by integrating their advantages. Notably, the Local stream achieved higher Specificity than the Global stream, revealing the latter’s limitation in leveraging inter-class distinctions.

**TABLE 5:** Comparative results of the proposed network and its subnetworks (Global flow and Local stream).

Network	SPEC	SENS	ACC
Global Stream	0.8950	0.8882	0.8858
Local Stream	0.9050	0.7866	0.8281
**Dual-stream ELNet**	**0.9650**	**0.8882**	**0.9104**

Bold values indicate the results of the proposed dual-stream ELNet.

**FIGURE 6:**
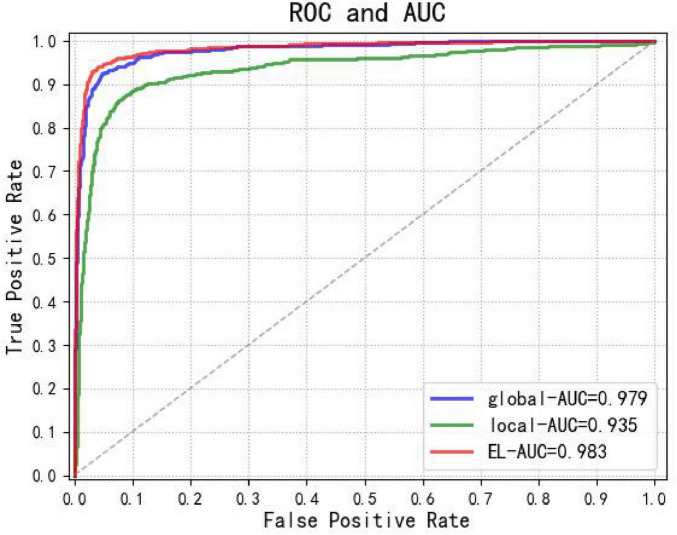
ROC curves and AUC values of the dual-stream ELNet (red), Global Network (blue), and Local Network (green).

Figure 6 presents the Area Under the Curve (AUC) and Receiver Operating Characteristic (ROC) analysis for the Dual-stream ELNet and its subnetworks, demonstrating that the proposed algorithm outperforms both standalone local (AUC: 0.935) and global (AUC: 0.979) networks.

Table 6 shows the Accuracy (ACC), Positive Predictive Value (PPV), and Negative Predictive Value (NPV) for four esophageal image types, with ACC values of 94.07% (normal), 96.39% (esophagitis), 98.55% (heterotopic mucosa), and 93.06% (cancer). Esophageal cancer had the highest misdiagnosis rate.

**TABLE 6:** ACC, PPV, and NPV of the dual-stream ELNet network for normal, inflammatory, heterotopic mucosa, and cancerous conditions.

Evaluation metric	Normal	Inflammation	Heterotopic mucosa	Esophageal cancer
ACC	94.07%	96.39%	98.55%	93.06%
PPV	96.5%	94.68%	95%	95.35%
NPV	93.09%	97.02%	99.15%	92.31%

### 3.2 Segmentation of esophageal lesions

This study used SNC to create three structurally identical U-Net networks for segmenting esophageal lesions: esophagitis, gastric heterotopia, and cancer. Table 7 shows SNC and SNNC quantitative results for esophageal lesion segmentation. SNC effectively distinguishes the three lesion types and reduces false negatives and positives. In contrast, SNNC has higher error rates due to the lesions’ distinct locations, colors, and shapes.

**TABLE 7:** Quantitative segmentation results of esophageal lesions by SNC and SNNC (SNNC results in parentheses).

Evaluation metric	Inflammation	Esophageal cancer	Heterotopic mucosa	Mean
ACC	**0.9282**	**0.9075**	**0.9915**	**0.9462**
	(0.8806)	(0.7676)	(0.9152)	(0.8766)
SENS	**0.6909**	0.8020	**0.9387**	**0.8018**
	(0.5824)	**(0.8455)**	(0.5315)	(0.5838)
SPEC	**0.9648**	**0.9337**	**0.9954**	**0.9655**
	(0.9095)	(0.7374)	(0.9462)	(0.8968)

Bold values indicate the superior value between SNC and SNNC.

**FIGURE 7:**
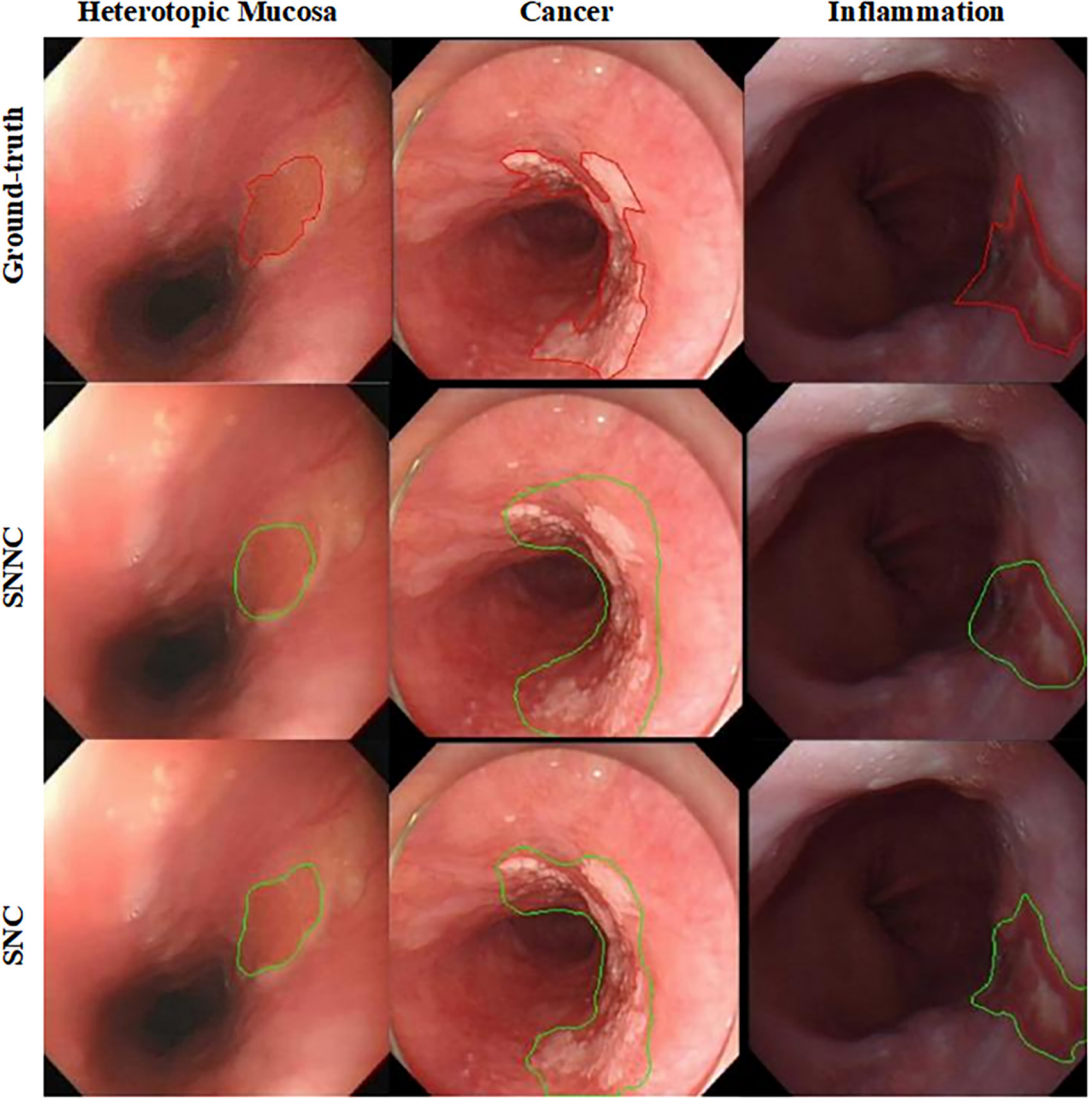
Qualitative segmentation results of SNC and SNNC for three types of esophageal lesions (Ectopia, Cancer, and Inflammation). The upper panel displays the ground truth highlighted with red contours, while the lower panel shows the predicted masks (SNC and SNNC) outlined in green. The three columns represent the three distinct types of esophageal lesions respectively.

Figure 7 shows qualitative segmentation results for three esophageal lesions by SNC and SNNC. SNC results agree well with expert manual delineations and reduce false positives/negatives due to strong adaptability. Conversely, SNNC has a higher false positive rate for cancer and esophagitis, mainly from insufficient data fitting.

## 4 Discussion

The primary aim was to enhance diagnostic accuracy, reduce miss rates, and provide a standardized, scalable tool to support endoscopists in clinical practice. Our findings demonstrate that the proposed model achieves high overall accuracy, with a specificity of 97.1% and sensitivity of 88.74%, indicating its potential as a reliable adjunctive tool in esophageal cancer screening. In this discussion, we interpret these results in depth, contextualize them within the existing literature, address key limitations, and propose actionable directions for future research.

First, we reaffirm the core findings of our study. The model successfully identifies most malignant lesions while maintaining a low false-positive rate, as reflected in its high specificity. This suggests strong discriminative capability between cancerous and non-cancerous tissues under white-light imaging conditions. Importantly, this performance is enabled by our clinically inspired cascaded design: rather than analyzing the entire image at once, we first localize suspicious regions using Faster R-CNN, which effectively filters out confounding structures such as normal mucosa, bubbles, and mucus. This focused analysis significantly reduces background interference and enhances the reliability of downstream classification and segmentation. Nevertheless, the sensitivity remains moderately lower than the specificity, which warrants further exploration.

Second, several factors may account for this discrepancy. One plausible explanation is environmental interference—despite our use of the Navier-Stokes method, residual mucus, bubbles, or strong specular highlights were not completely eliminated in certain cases, leading to misclassification. Another critical factor is intra-class variability. Specifically, our analysis reveals the highest misdiagnosis rate among subtle lesions characterized by mild erythema or slight surface irregularity. These ambiguous presentations are often indistinguishable even to experienced endoscopists, and due to their low prevalence in the training set, the model lacks sufficient exposure to learn robust features for such edge cases. This data imbalance likely contributes to the reduced sensitivity and highlights the challenge of generalizing AI models to atypical or early-stage presentations.

Third, when comparing our results with prior studies, our work both aligns with and extends existing knowledge. Previous CNN-based models in gastrointestinal endoscopy have reported sensitivities ranging from 85 to 93% and specificities above 95%, depending on dataset composition and imaging modality (2). While our sensitivity falls within this range, our model distinguishes itself through its focus on real-world clinical applicability—specifically, its integration into routine white-light endoscopy without requiring specialized equipment. Unlike many studies that rely on narrow-band imaging (NBI) or magnified endoscopy, our system operates on widely available standard-definition white-light images, increasing its accessibility across diverse healthcare settings. Furthermore, by embedding preprocessing modules directly into the pipeline, we enhance robustness against common image degradation issues, representing a practical advancement over previous frameworks that assume ideal image quality.

Fourth, it is important to acknowledge the limitations of the current study. First, the dataset size is relatively limited, comprising 789 annotated images from a single institution. Although data augmentation was applied, the sample may still be insufficient to capture the full spectrum of lesion morphologies, particularly rare subtypes or early neoplastic changes. Second, the reliance on white-light imaging inherently constrains the model’s ability to detect microvascular patterns critical for differentiating superficial neoplasia—a limitation that cannot be fully overcome by architectural improvements alone. Third, the retrospective design introduces potential selection bias, and external validation on multi-center, diverse populations is needed to assess generalizability. Fourth, the current framework processes each task sequentially (localization → classification → segmentation), which, while clinically intuitive, does not allow for iterative refinement between stages. Future work could explore feedback mechanisms or joint optimization to further improve coherence across tasks.

Fifth, based on these limitations, we propose several avenues for future research and methodological refinement. To address data scarcity, semi-supervised learning frameworks—such as FixMatch or Noisy Student Training—could be employed to leverage large volumes of unlabeled endoscopic images, thereby enhancing model generalization without prohibitive annotation costs. Additionally, integrating attention mechanisms (e.g., CBAM or SE blocks) into the ELNet architecture may improve feature discrimination by allowing the model to dynamically focus on salient regions while suppressing background noise, potentially boosting sensitivity. More importantly, future work should explore multi-modal data fusion strategies, combining white-light images with NBI or magnified endoscopy to provide complementary information about mucosal and vascular architecture. Such an approach holds promise for significantly improving early detection rates, especially for flat or indistinct lesions.

## 5 Conclusion

Worldwide, esophageal cancer is regarded as a leading type of cancer affecting the gastrointestinal system. In China, a high-incidence region, both mortality and morbidity rates remain relatively elevated. Therefore, early detection is crucial (21). Advancing endoscopic techniques and health education have increased early esophageal cancer detection. However, many hospitals lack trained endoscopists, leading to variable examination quality. In Japan, EP-SM1 esophageal carcinoma is treated with endoscopic resection, while SM2 cases require chemoradiotherapy or surgery, making preoperative invasion depth assessment critical.

In recent years, deep learning methods have found increasingly extensive applications in the medical field, demonstrating particularly superior performance in the recognition of medical images. A study demonstrates that integrating multimodal data with quantum-enhanced technology achieves high accuracy (96%) and high sensitivity (94%) in glaucoma diagnosis, while significantly reducing computational complexity and enhancing the efficiency and practicality of clinical deployment (22). Tokai et al. in Japan employed an artificial intelligence diagnostic system to detect superficial esophageal squamous cell carcinoma in endoscopic images (23). While assessing its invasion depth, the system also achieved high detection accuracy. This AI diagnostic system achieved a specificity of 73.3%, sensitivity of 84.1%, and accuracy of 80.9% in diagnosing EO-SM1 lesions. These findings indicate that the diagnostic accuracy of artificial intelligence far exceeds that of most endoscopists, while also operating at significantly faster speeds. The convolutional neural network framework proposed in this study can serve as a secondary endoscopist during endoscopic examinations, enabling real-time segmentation and classification of esophageal lesions in endoscopic images. This approach effectively reduces missed diagnosis rates and provides clearer delineation of lesion extent. Experimental results demonstrate that the dual-stream ELNet architecture significantly outperforms both local and global stream networks in esophageal lesion classification. Statistical analysis using AUC and ROC curves shows the dual-stream ELNet achieves an accuracy of 0.9104, specificity of 0.9650, and sensitivity of 0.8822. Classification accuracy rates reached 93.06% for esophageal cancer, 98.55% for esophageal gastric heterotopia, 96.39% for esophagitis, and 94.07% for normal esophagus—all exceeding 90%. These results confirm the model’s robust classification performance. Esophageal cancer exhibited the highest misdiagnosis rate. The lesions exhibited inconspicuous features—manifesting only as slight redness or mild roughness of the esophageal mucosa—making them exceptionally difficult to detect.

While endoscopic treatment techniques have advanced rapidly, Lugol’s iodine staining remains the primary method for delineating esophageal lesion boundaries. A key highlight of this study lies in employing the U-Net network to directly segment esophageal lesions while simultaneously determining their extent. The segmentation achieved accuracy, specificity, and sensitivity values of 0.9435, 0.9536, and 0.8093, respectively. In the latest study, Guo et al. developed a computer-aided diagnosis system (CAD) trained on narrow-band imaging images and validated it through video datasets (24). The system demonstrated a frame-wise sensitivity of 100% for each lesion in every frame of the magnified videos, attaining an overall sensitivity of 96.4%. Additionally, de Groof AJ et al. created a hybrid ResNet-UNet model CAD system, achieving 89% accuracy, 90% sensitivity, and 88% specificity in classifying neoplastic versus non-neoplastic Barrett’s esophagus (25). In another dataset, the CAD system outperformed all 50 participating endoscopists in specificity, sensitivity, and accuracy rates. Real-time CAD systems hold significant promise for future clinical applications by assisting endoscopists in diagnosing early-stage esophageal cancer and precancerous lesions.

The key contributions of this study include: First, establishing an esophageal endoscopic image database comprising 789 white-light images. We divided these into four distinct categories: normal esophagus, esophagitis, esophageal gastric heterotopia, and esophageal cancer; each image contains corresponding classification labels and segmentation annotations. Second, developing a dual-stream ELNet network for automated classification of three esophageal lesion types—esophagitis, esophageal cancer, esophageal gastric heterotopia—and normal esophagus. The designed framework employs a dual-path architecture, comprising a global flow and a local stream. The local stream focuses on extracting local features of lesion patches through a Faster-RCNN detector, while the global flow extracts global features from the entire esophageal image. These two streams are mutually integrated to classify four types of esophageal images. Thirdly, based on the classification results from Dual-stream ELNet, corresponding U-Net networks are employed to segment three types of esophageal lesions, ultimately localizing the esophageal lesion sites.

In summary, our work introduces a clinically inspired cascaded framework that mirrors the real-world diagnostic workflow: suspicious regions are first localized via Faster R-CNN to suppress background interference from non-lesion structures (e.g., bubbles, mucus, normal mucosa); the localized region is then classified into one of four categories using the dual-stream ELNet, which fuses global context and local discriminative features; finally, the classification outcome dynamically routes the input to a corresponding category-specific U-Net for precise segmentation. Crucially, only one U-Net is activated during inference, ensuring computational efficiency without sacrificing accuracy. Evaluated on 789 annotated white-light endoscopic images, the system achieves over 93% classification accuracy across all categories and 80.93% segmentation sensitivity—demonstrating strong performance even for subtle early cancers. This structured, task-specialized pipeline offers a practical and scalable solution for real-time, AI-assisted esophageal lesion analysis in routine clinical endoscopy.

Future work on the algorithm proposed in this study primarily includes: first, constructing a new network framework for more refined classification of esophageal cancer, such as distinguishing between esophageal squamous cell carcinoma and adenocarcinoma (26); second, implementing semi-supervised CNN for esophageal lesions when using larger training databases, addressing the scarcity of classification labels and annotations. Finally, developing a novel network framework to classify and segment common lesions throughout the upper gastrointestinal tract while delineating lesion boundaries.

## Data Availability

The raw data supporting the conclusions of this article will be made available by the authors, without undue reservation.
